# Oesophageal balloon positioning by echocardiography to guide positive pressure ventilation

**DOI:** 10.1007/s10877-021-00730-z

**Published:** 2021-06-20

**Authors:** Marco Betello, Raphael Giraud, Karim Bendjelid

**Affiliations:** 1 Anesthesiology Division, Geneva University Hospitals, Rue Gabrielle-Perret-Gentil 4, 1211 Genève 4, Switzerland; 2 Intensive Care Division, Geneva University Hospitals, Geneva, Switzerland; 3 Geneva Hemodynamic Research Group, Geneva, Switzerland; 4 Faculty of Medicine, Geneva, Switzerland

**Keywords:** Ultrasounds, Oesophageal balloon, Chest wall compliance, COVID-19, ARDS, Transpulmonary pressure

## Abstract

Understanding the respiratory mechanics of ARDS patients is crucial to avoid ventilator-induced lung injury (VILI), and this is much more challenging if not only lung compliance is altered but the whole compliance of the respiratory system is abnormal, as in obese patients. We face this problem daily in the ICU, and to optimize ventilation, we estimate respiratory mechanics using an oesophageal balloon. The balloon position is crucial to assess reliable values. In the present technical note, we describe the use of echocardiography to confirm the correct position of this instrument.

## Introduction

In terms of ventilatory mechanics, patients with ARDS have low pulmonary compliance. Mechanical ventilation of these patients with a “baby lung” is challenging [1], and a subtle ventilator setting is needed. In this regard, monitoring the normalized tidal volume to the compliance of the respiratory system (driving pressure) is a way to optimize ventilation settings to limit the occurrence of ventilator-induced lung injuries (VILIs) [2, 3].

Driving pressure (ΔP) is the difference between plateau pressure and PEEP and can be influenced by changes in tidal volume (VT), PEEP, intrinsic PEEP or respiratory system compliance. Lowering VT for a constant PEEP value decreases ΔP. Similarly, increasing the PEEP value for a constant VT value can also decrease ΔP if significant pulmonary recruitment occurs and lung compliance improves. A retrospective analysis demonstrated that ΔP is the variable that is most strongly associated with mortality in ARDS and that lower ΔP is associated with lower mortality [2]. In addition, a recent meta-analysis confirmed an association between higher ΔP and higher mortality in mechanically ventilated patients with acute respiratory distress syndrome [4].

A major limitation of ΔP is its dependence on the properties of the whole respiratory system and not exclusively the lungs. Indeed, whereas ΔPs are easier to assess for guidance to avoid ventilator-induced lung injury, there are limitations. External to the lungs, the properties of the chest wall, including the abdomen, influence ΔP measurements. This influence could be misleading, as chest wall properties do not reflect an increased risk of injury [5]. When chest wall compliance is abnormal (e.g., post cardiac surgery, chest radiotherapy, high BMI) or variable (e.g., ascites, abdomen surgery), direct assessment of transpulmonary pressure (plateau pressure minus pleural pressure) could be required to appropriately quantify potentially damaging stress applied to the lungs. In this regard, the insertion of an oesophageal balloon to measure mediastinal pressure and calculate transpulmonary pressures may help to set the optimal plateau pressure and PEEP values in mechanically ventilated critically ill patients with severe ARDS. Indeed, transpulmonary pressure is used most frequently in the intensive care unit to guide the PEEP setting in the most difficult patients, including patients with ARDS and obese patients [6].

Compared with the current standard of care, a ventilator strategy using oesophageal pressure (Pes) measurements to estimate transpulmonary pressure significantly improves oxygenation and compliance in patients with ARDS without inducing VILI [7]. The present monitoring is easily feasible using an oesophageal balloon. However, the present technique is less commonly used in clinical practice, probably because of concern about the correct positioning and interpretation of pressure curves. There is a close association between the reliability of Pes values to assess pleural pressure and the balloon position in the mediastinum.

## Background: checking the good position

**Fig. 1:**
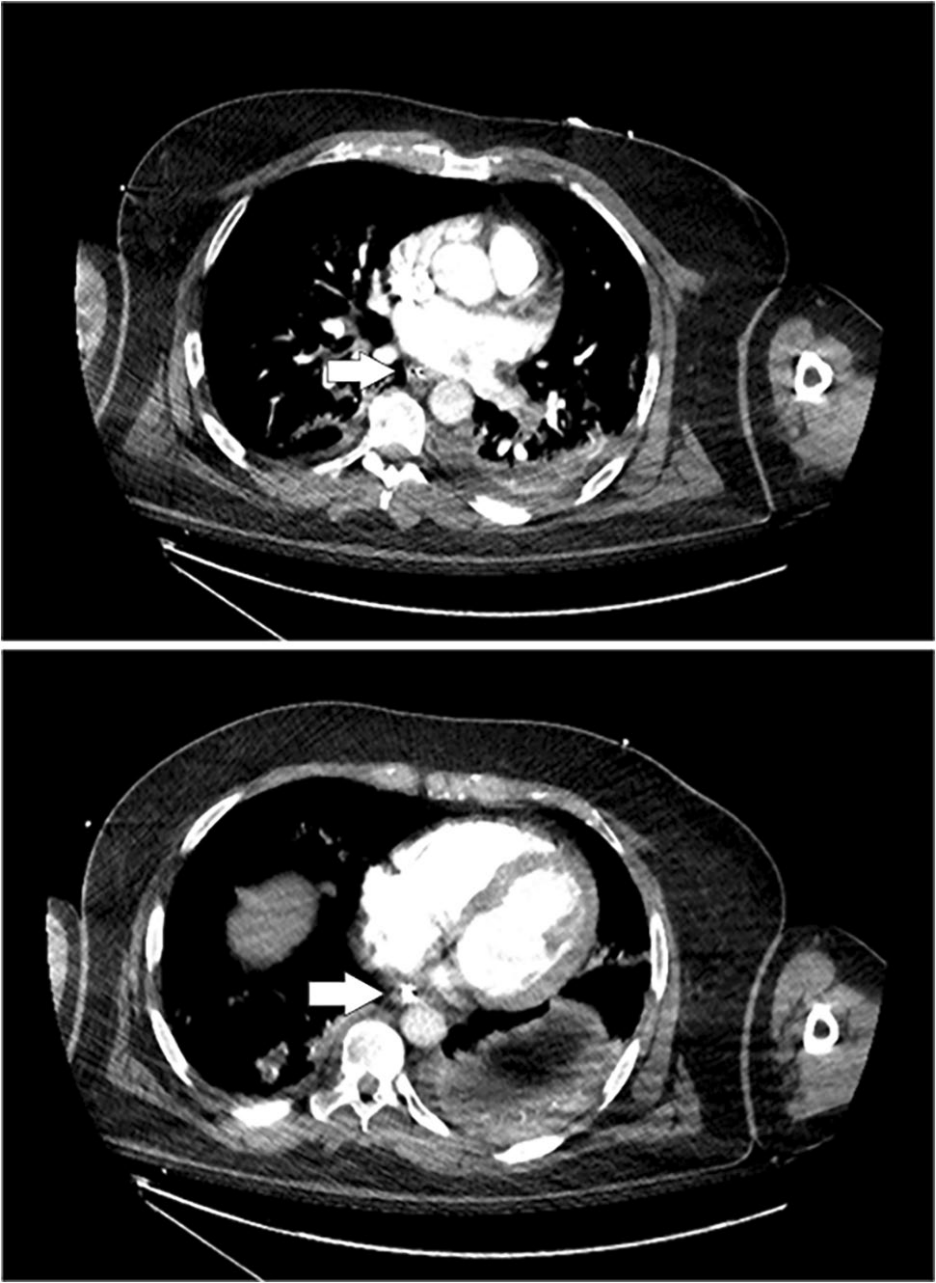
Tomographic visualization of the esophageal balloon showing its strict relation with the posterior wall of the left atrium

The first step to obtain a reliable measure of the oesophageal pressure is to ensure the good position of the balloon. The upper oesophageal position (first third) should be avoided because of the close relationship between the trachea and the oesophagus that leads to a directed transmission of upper airway pressures to the balloon. The device should, therefore, be advanced to the second one-third of the oesophagus, as in the third one-third oesophagus position, pressure measurements are disturbed by cardiac oscillations. Additionally, in patients in the supine position, the gravitational mediastinal compression of the balloon (compliance alteration) observed in the third one-third of the oesophagus can be avoided [8–10]. The good position is normally established indirectly by introducing the balloon in the stomach. The device is then inflated and withdrawn to obtain cardiac oscillation and pressure variations synchronous with ventilation. The proper position is then confirmed by occlusion tests. If doubts on the position persist, most catheters have a radiopaque marker, and an X-ray or a CT scan (Fig. 1) could be performed to check the right position [11]. In the present technical note, we describe an ultrasound method of direct and indirect visualization of oesophageal balloon in an adequate intrathoracic position that could be used to assist in assessing the right balloon position.

## The ultrasounds technique

The mid-low oesophagus lies behind the heart in direct contact with the left atrium; its lowest part, just before passing through the diaphragm, is in proximity to the left/right ventricle. Normally, it has a virtual lumen and is not visible by ultrasound unless dilated or occupied by an expansion lesion. In this regard, there are several clinical reports describing transthoracic echocardiographic visualization of a dilated oesophagus with compression of the left atrium [12].

**Fig. 2:**
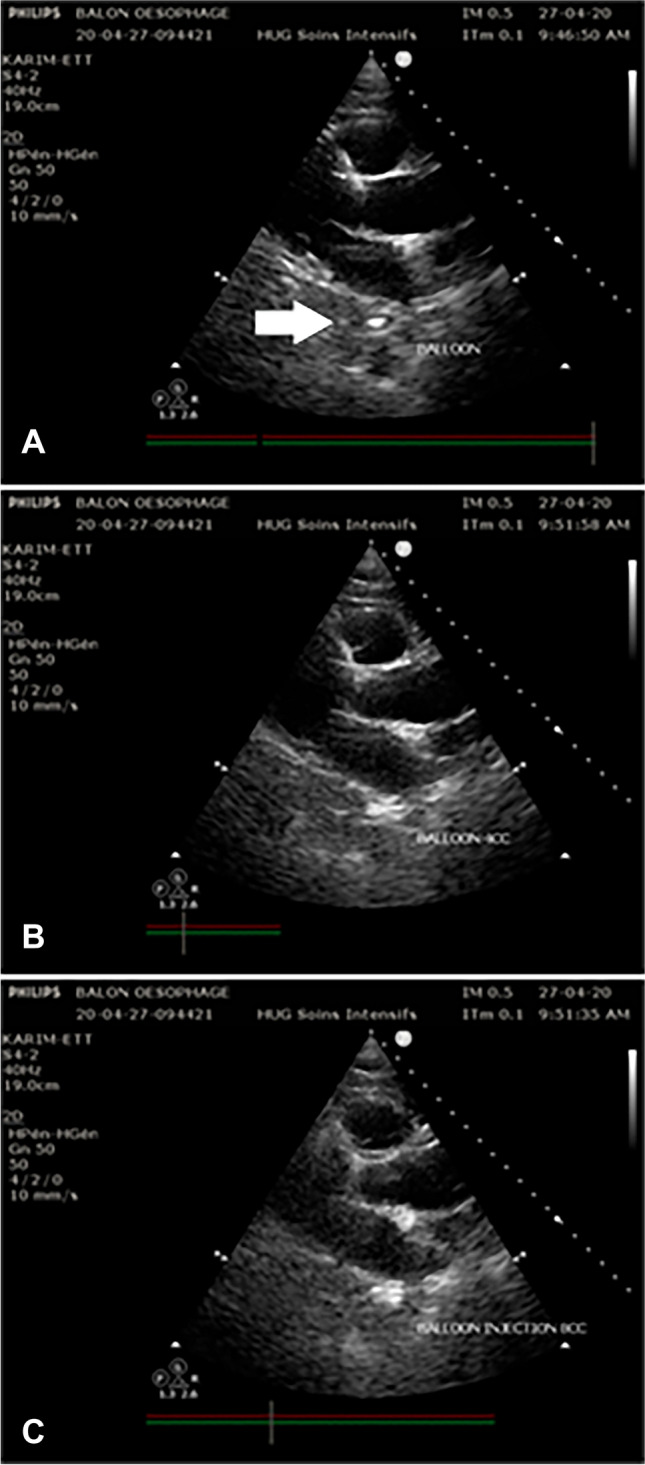
Direct visualization of esophageal balloon by PLAX window: **A** Visualization of the nasogastric tube as an hyperechogenic dot (white arrow) between the left atrium and the thoracic aorta; **B** and **C** After the injection of respectively 4 cc and 8 cc of air in the esophageal balloon the image of the hyperecogenic dot is replaced by à hypercogenic line and the image of the thoracic aorta disappeared because of ecographic shadowing by the balloon

**Fig. 3:**
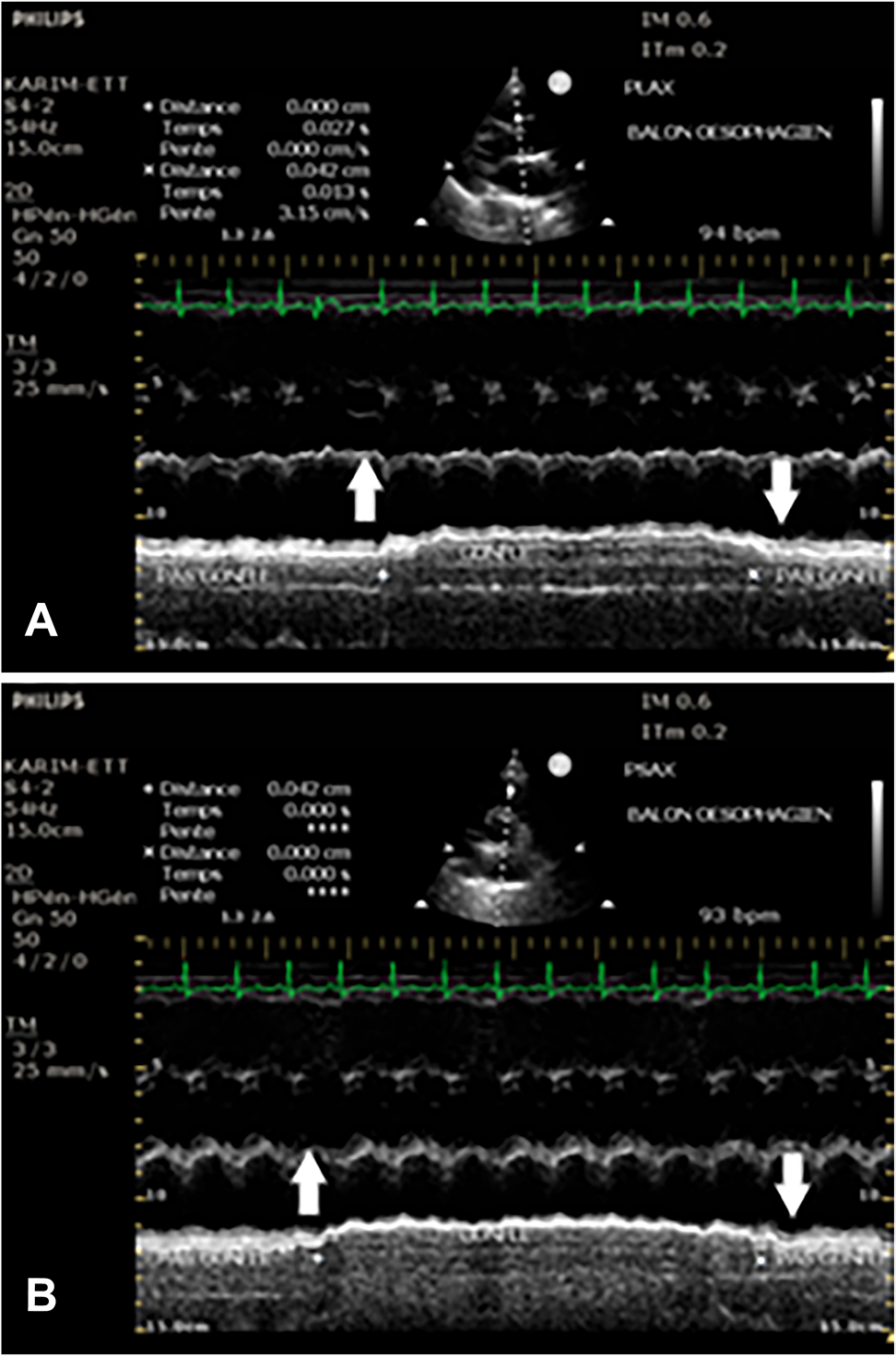
Indirect visualization of esophageal balloon by M-Mode in parasternal windows: **A** PLAX with M-Mode aiming the posterior wall of the left atrium. After the injection of 4 cc of air in the esophageal balloon we note an anterior displacement of the posterior wall of the atrium (upwards arrow) marking the presence of the esophageal balloon behind the atrium. After deflating the balloon we note the line of the posterior wall of atrium returning to the original position (downwards arrow); **B** The same method to visualize the balloon is used in PSAX showing the same reversible anterior displacement of the posterior wall of the atrium

**Fig. 4:**
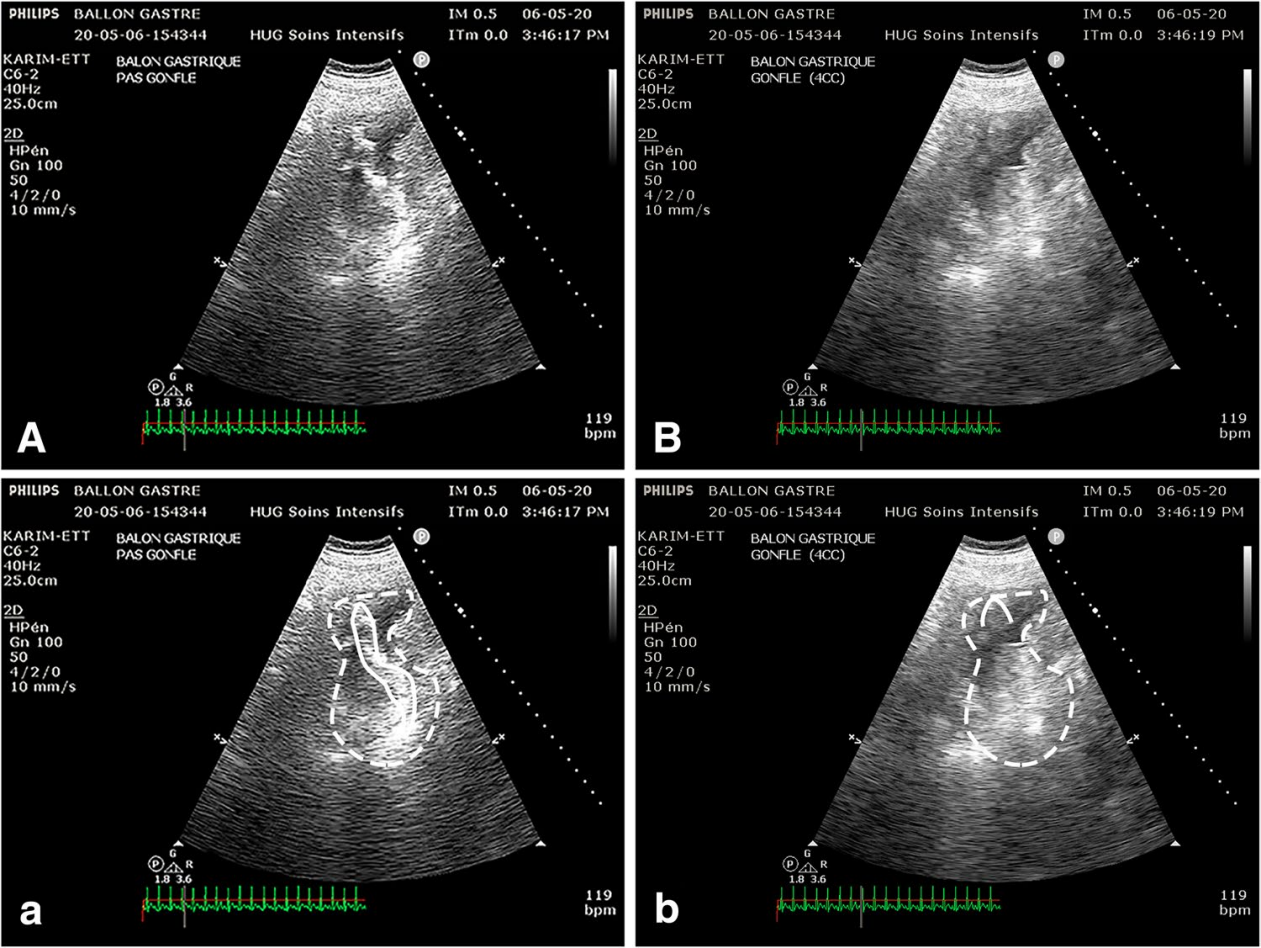
Direct visualization of balloon in gastric position by subxifoid window: **A**, **a** Image of the stomach (dashed line) with hyperechoic linear image that correspond to the balloon (solid line); **B**, **b** After injecting 4 cc of air in the balloon the linear hyperechoic image disappear and is remplaced by a hypercoic line with posterior shadowing confirming that the image correspond to the balloon that is misplaced in gastric position

In the present technical note, the reader will find figures that display how the balloon catheter could be directly discerned by echocardiography in the parasternal long axis (PLAX) and parasternal short axis (PSAX) behind the left atrium and that inflating the balloon causes a change in the 2D image (Fig. 2). Nevertheless, if the catheter is not visualized in 2D, indirect visualization is possible using the M-Mode at the level of the left atrium that will show proximal displacements of the posterior wall of the atrium (anterior wall of the oesophagus) when the balloon is inflated, allowing for the witnessing of the close relationship between the two structures (Fig. 3). The reader will also find images of one of the cases in which the balloon was not directly or indirectly visualized in the PLAX because it was inserted too far, and the ultrasound technique permitted the medical professional both to see the balloon inside the gastrum (Fig. 4) and to correct the position in the oesophagus.

## Data Availability

Not applicable.
